# Regulation of RNA-binding proteins affinity to export receptors enables the nuclear basket proteins to distinguish and retain aberrant mRNAs

**DOI:** 10.1038/srep35380

**Published:** 2016-11-02

**Authors:** M. Soheilypour, M. R. K. Mofrad

**Affiliations:** 1 Molecular Cell Biomechanics Laboratory, Departments of Bioengineering and Mechanical Engineering, University of California, Berkeley, CA, 94720, USA; 2 Molecular Biophysics and Integrative Bioimaging Division, Lawrence Berkeley National Laboratory, Berkeley, CA, 94720, USA

## Abstract

Export of messenger ribonucleic acids (mRNAs) into the cytoplasm is a fundamental step in gene regulation processes, which is meticulously quality controlled by highly efficient mechanisms in eukaryotic cells. Yet, it remains unclear how the aberrant mRNAs are recognized and retained inside the nucleus. Using a new modelling approach for complex systems, namely the agent-based modelling (ABM) approach, we develop a minimal model of the mRNA quality control (QC) mechanism. Our results demonstrate that regulation of the affinity of RNA-binding proteins (RBPs) to export receptors along with the weak interaction between the nuclear basket protein (Mlp1 or Tpr) and RBPs are the minimum requirements to distinguish and retain aberrant mRNAs. Our results show that the affinity between Tpr and RBPs is optimized to maximize the retention of aberrant mRNAs. In addition, we demonstrate how the length of mRNA affects the QC process. Since longer mRNAs spend more time in the nuclear basket to form a compact conformation and initiate their export, nuclear basket proteins could more easily capture and retain them inside the nucleus.

Following transcription, messenger ribonucleic acids (mRNAs) are transported to the cytoplasm to transfer genetic information and direct synthesis of functional proteins^1^. Multiple co-transcriptionally occurring processes applied on precursor mRNA (pre-mRNA) are followed by the engagement of several key proteins and complexes, including RNA-binding proteins (RBPs), along the length of pre-mRNA^2^,^3^, eventually forming an export-competent ribonucleoprotein (mRNP) prepared for efficient export through the nuclear pore complex (NPC). Splicing, 5′ capping, 3′ cleavage and polyadenylation are the four well-known processing steps prior to nuclear export, while failure in any of these steps yields aberrant mRNAs^1^,^3^. These processes are quality controlled by various evolutionary conserved and highly efficient mechanisms in eukaryotic cells^3^,^4^. mRNA quality control (QC) occurs at different stages of RNA biogenesis^5^ and is primarily dependent on intra-nuclear protein-protein interactions, wherein RNA-binding proteins (RBPs) play a major role^6^,^7^. Proteomic studies have suggested that lack of RBPs may result in different diseases such as cancer and neurodegenerative conditions^8^.

Several experimental studies have identified a multitude of proteins (such as nuclear basket associated protein Mlp1 (Tpr in human) and RBPs such as Nab2, Npl3, Gbp2, and Hrb1) that play a role in mRNA QC as their deletion or mutation in their genes lead to the leakage of pre-mRNAs into the cytoplasm^9^,^10^,^11^,^12^,^13^,^14^,^15^,^16^,^17^,^18^,^19^. Although most of these studies have been focused on yeast mRNA QC mechanism, similar proteins and complexes are identified in human^20^,^21^,^22^. However, despite the many studies conducted to understand the molecular events involved in mRNA QC processes, the exact mechanism through which mRNAs are recognized as incorrect remains elusive^5^,^19^. More specifically, it is still unknown what are the minimum factors and interactions that are required to distinguish and retain aberrant mRNAs.

The major proteins involved in QC of mRNAs at the nuclear basket of the NPC can be categorized into two groups, 1) nuclear basket-associated proteins and 2) RBPs that are directly bound to mRNAs. The nuclear basket-associated proteins include Pml39, Tpr in vertebrates or Mlp1 and Mlp2 in yeast, and Nup60. Of these, Tpr (Mlp1) is suggested to play a key role in retention of aberrant mRNAs^11^,^12^,^19^,^23^,^24^,^25^. Mlp1 and Mlp2, homologs of Tpr in *Saccharomyces cerevisiae*, are not essential for cell growth and have been primarily suggested to possess a redundant function^26^,^27^. Overexpression of Mlp1 is associated with nuclear accumulation of mRNA^28^ and its deletion leads to leakage of pre-mRNAs^10^. Different research groups characterized the interaction between Mlp1 and different RBPs and proposed Mlp1 as a checkpoint in the nuclear basket, which evaluates the maturity of passing mRNPs^19^,^23^,^24^. Tpr is also suggested to possess the same role in mRNA QC^11^,^12^.

RBPs, as the second category of proteins involved in mRNA QC, are identified as recruiters of the export receptor heterodimer NXF1/NXT1 (Mex67/Mtr2 in yeast). Npl3^29^, Nab2^30^, Gbp2 and Hrb1^19^ in yeast, and 9G8, SRp20, and ASF/SF2 in vertebrates^31^ are the identified RBPs involved in this process. Other proteins, such as Yra1 and its human homologue Aly, are also suggested to regulate the recruitment of export receptors to mRNAs^30^,^32^,^33^. However, they are found to be dispensable for mRNA export and act more as cofactors for stabilization of the interaction between RBPs and the export receptor^30^.

Therefore, the current proposed model for mRNA export and QC at the nuclear basket^3^,^19^,^30^ suggests RBPs as adapters for recruitment of export receptor heterodimers (presumably in concert with other elements as cofactors), while, upon initiation of mRNA export through the NPC, Tpr (Mlp1) interacts with RBPs, acting as a checkpoint to verify the maturity of mRNP for export^19^,^23^,^24^ (Fig. 1). Nevertheless, it is still elusive how the collective behaviour of these factors and the kinetics of their interactions could efficiently distinguish aberrant and normal mRNAs.

The lack of detailed understanding of mRNA QC mechanism could be partly attributed to the complex nature of this process, which makes it not easily tractable via experimental and conventional computational approaches. As a result, we employ a complex systems modelling approach, namely agent-based modelling (ABM)^34^,^35^,^36^, to explore mRNA QC mechanism (Fig. 2). We seek to develop a minimal model for mRNA QC and identify the minimum required factors to efficiently distinguish and retain aberrant mRNAs. We hypothesize that the two abovementioned categories of the involved factors, namely RBPs and the nuclear basket associated protein Tpr (Mlp1 in yeast), are the major role-players in this process. Accordingly, we design a systematic procedure to demonstrate whether cross-interactions of these proteins could lead to proper retention of aberrant mRNAs. It should be noted that our model is, by no means, a comprehensive representation of the mRNA export and QC system, due to several unknowns that still exist (limitations of the model is thoroughly discussed in the Supplementary Information – section A). Instead, using the available experimental data, we seek to identify the minimal system that is able to distinguish and retain aberrant mRNAs. Our computational models, along with recent single-molecule imaging techniques^37^,^38^, could unveil valuable details of mRNA metabolism with a high spatiotemporal resolution.

## Results

We seek to test our hypothesis that the interactions between three types of proteins involved in mRNA export and QC, i.e. the export receptor, RBPs, and Tpr, are the key to distinguish aberrant mRNAs. Accordingly, we take a step-wise approach: first, the interaction between Tpr and RBPs is inhibited in the model to evaluate whether only regulation of the interaction between RBPs and export receptors is sufficient to retain aberrant mRNAs or not. We evaluate this system under different environmental conditions for different configurations of mRNAs. Subsequently, in the next step, we implement the interaction between Tpr and RBPs.

### Is regulation of the interaction between RBPs and the export receptor sufficient to retain aberrant mRNAs inside the nucleus?

Splicing is not an absolute requirement for the indirect interaction of export receptors with RNA^39^,^40^, and the export receptor in metazoans (TAP) has a higher affinity to RBPs bound to spliced mRNAs, compared to RBPs bound to unspliced mRNAs^41^. Therefore, RBPs bound to aberrant mRNAs could still recruit export receptors, yet with a lower affinity. In order to evaluate whether regulation of the interaction between RBPs and the export receptor is sufficient to retain aberrant mRNAs, we conducted a set of simulations on export of mRNAs with different affinities of RBPs to NXF1/NXT1. Two mRNA sequences of length 2.2 kb with different number of export receptor binding sites (9 and 12) are studied. Nuclear population of NXF1/NXT1 is varied across simulations, while the affinity of RBPs to export receptor is normal, reduced by 10-fold, or reduced by 100-fold. Based on experimental observations, mRNAs that are not fully processed (e.g. not spliced) are modelled as transcripts that have RBPs with a lower affinity to the export receptor^41^. Hereafter, these mRNAs are called *aberrant* mRNAs, while normally processed mRNAs that have RBPs with normal affinity to NXF1/NXT1^42^ are termed *normal* mRNAs.

Considering that a typical mRNA of 2.2 kb length is expected to have an average of 9 binding sites for the export receptor^43^, our first set of simulations is performed on an mRNA sequence with 9 RBPs (as binding sites for the export receptor). Percentage of successful mRNA export events for different expression levels of the export receptor within the nucleus is shown in Fig. 3. Higher concentration of the export receptor slightly increases the export of both the normal (Fig. 3, gray plot) and the aberrant mRNA (Fig. 3, pink plot). Export of aberrant mRNA reaches a plateau at approximately 7%, while export of normal mRNA slightly increases and reaches ~20%, which is in agreement with previously reported computational and *in vivo* export rates^36^,^44^. On average, the percentage of successful export events of normal mRNA is three times that of the aberrant mRNA (Fig. 3-boxplot). Therefore, a lower affinity of RBPs (bound to aberrant mRNAs) to the export receptor leads to a considerable retention of aberrant mRNAs, while allowing normal mRNAs to be exported efficiently. However, this should be further evaluated for other configurations of mRNAs as well.

Second set of simulations was conducted on an mRNA sequence of the same length but higher number of export receptor binding sites (12 instead of 9). Percentage of successful export events with respect to the population of export receptor using different affinities between RBPs and NXF1/NXT1 is shown in Fig. 4. As can be seen, the behaviour is significantly different from the previous experiment. The normal mRNA yields a successful export percentage of ~83%, in agreement with our previous mRNA export study (Fig. 4, gray plot)^36^. Interestingly, regardless of NXF1/NXT1 copy number (except for the first data point), lowering the affinity of RBPs to NXF1/NXT1 by 10-fold for aberrant mRNAs (Fig. 4, pink plot) would not inhibit efficient mRNA export, resulting in the same percentage of export events as the normal mRNA (Fig. 4-boxplot). Nonetheless, further decrease of the affinity, i.e. 100-fold decrease (see Fig. 4, cyan), substantially decreases the export rate, regardless of export receptor copy number. Collectively, our results demonstrate that while regulation of the affinity between RBPs and the export receptor (via phosphorylation of the RBPs) could retain aberrant mRNAs with average number of export receptor binding sites, it is not sufficient to retain all configurations of aberrant mRNAs.

### Lower affinity of RBPs to the export receptor enables Tpr to distinguish and retain aberrant mRNAs

As the next step in developing our minimal model of the mRNA QC, the interaction between Tpr and RBPs is included in the model. An mRNA of length 2.2 kb with 12 RBPs (as the configuration that resulted in the largest number of aberrant mRNA export events in the absence of the interaction between Tpr and RBPs (Fig. 4)) is simulated. The percentage of successful export events of the mRNA is shown in Fig. 5. Interestingly, regardless of the population of NXF1/NXT1, the weak interaction between Tpr and RBPs efficiently distinguishes aberrant mRNAs and prevents their export (Fig. 5, pink bars), while allowing normal mRNAs to be exported properly (Fig. 5, gray bars). This confirms our hypothesis that a lower affinity of RBPs to the export receptor, along with the interaction between Tpr and RBPs is the key to hinder the export of aberrant mRNAs.

### mRNA QC is a length-dependent mechanism

In order to understand whether mRNA length plays a role in the QC process, we repeated the previous study by simulating a shorter mRNA sequence of length 500 b with the same density of RBPs (number per unit length) along its length. The percentage of export events of the mRNA with respect to NXF1/NXT1 copy number is presented in Fig. 6. Although aberrant mRNAs are relatively retained, the QC mechanism is not as efficient as it was in the case of longer mRNAs. Therefore, length of the mRNA is a determining factor in this process. Of note, normal mRNAs are still being exported efficiently.

### mRNA compaction at the nuclear basket is the reason for the length-dependency of the QC mechanism

Next, we sought to understand why mRNA length affects the QC process. The rate-limiting step in transport of mRNA molecules is suggested to be the at the nuclear basket, where the mRNA requires a considerable time to achieve an optimal configuration to initiate transport through the pore^36^,^44^,^45^. Therefore, since the quality control step is located at the nuclear basket, its length-dependent nature could be related to the difference in the optimal configuration of differently-sized mRNAs. Therefore, we conducted a set of simulations where two mRNAs of length 2.2 kb and 500 b (with the same density of RBPs) were simulated and their location and end-to-end distance were monitored throughout the simulation. The average end-to-end distance of both mRNAs is presented in Fig. 7 with respect to their distance from the central channel of the NPC. As the longer mRNA approaches the nuclear basket, it requires to form a more compact conformation to be able to thread into the NPC^36^, which is a time-consuming process. In contrast, the short mRNA does not show any compaction in size as it reaches the NPC. Measuring the residence time of the long and short mRNAs inside the nuclear basket also shows that the short mRNA spends about 35% less time compared to the long one. Therefore, one could conclude that since longer mRNAs spend more time at the vicinity and inside the nuclear basket to form an optimal configuration for export, Tpr has more time to check their maturity and reject them in the case of identifying an aberrant mRNA.

### The interaction between Tpr and RBPs is optimized for an efficient retention of aberrant mRNAs

In order to demonstrate the role of the interaction between Tpr and RBPs, we compared the export rate of aberrant mRNAs with respect to the change in the affinity of this interaction. Two different populations of export receptor (20 and 100 molecules) and two different lengths of mRNA (500 b and 2.2 kb) were simulated. The experimentally measured affinity of ~1 μM is optimized, as it is the weakest interaction that leads to the minimum export rate of aberrant mRNAs (in other words the highest retention of aberrant mRNAs). Higher affinities lead to the same rate and lower affinities result in considerably higher export rates of aberrant mRNAs (Fig. 8). Therefore, our results demonstrate that the interaction between Tpr and RBPs is optimized for the most efficient retention of aberrant mRNAs.

## Discussion

Despite numerous studies conducted to understand the mRNA QC procedure, the underlying mechanism is still unclear^19^. While several different proteins are identified to be involved in retention of aberrant mRNAs, no detailed explanation for how aberrant mRNAs are distinguished is presented so far. It is not even clear whether normal mRNAs are selected to be exported (selection model), aberrant mRNAs are retained inside the nucleus (retention model), or a combination of both strategies is employed in eukaryotic cells^46^.

In this work, we developed a computational model to identify the minimal system required to distinguish and retain aberrant mRNAs. Our results demonstrate that mRNA QC is primarily achieved by a cooperation between two key interactions: 1) the interaction between RBPs and the export receptor and 2) the interaction between the nuclear basket associated Tpr and RBPs. We showed that regulation of the interaction between RBPs and the export receptor enables Tpr to distinguish and retain aberrant mRNAs. The interaction between RBPs and the export receptor is regulated by their phosphorylation state. Npl3, one of the yeast RBPs, is phosphorylated when recruited to pre-mRNAs; and upon successful processing of mRNAs becomes dephosphorylated^47^. Hypophosphorylated form of 9G8 and ASF/SF2 (two metazoans RBPs) that is bound to normal mRNAs also have a higher affinity to the export receptor (TAP) compared to their hyperphosphorylated form that is bound to aberrant mRNAs^41^. Therefore, RBPs bound to aberrant mRNAs have lower affinity to the export receptor, compared to the RBPs bound to normal mRNAs. This slight difference, as demonstrated in this work, is sufficient for Tpr to distinguish and retain aberrant mRNAs. It was previously suggested that formation of aberrant mRNAs might be signalled via a yet-to-be-identified intron-bound protein to retain the mRNA^46^. However, our results suggest that mRNA QC is achieved by cooperation of regulated stochastic interactions between the involved proteins rather than deterministic switch-like properties. Nevertheless, the system we identified as the minimal requirement for mRNA QC is not perfect in retaining aberrant mRNAs, specifically for short mRNAs (Fig. 6). Therefore, the hypothesis that some yet-unknown proteins might signal the formation of aberrant mRNAs is still valid, which could further improve mRNA QC efficiency.

Our results highlight the effect of the density (number per unit length) of export receptor binding sites on mRNA sequence, and the length of the mRNA on mRNA export and QC. Regarding the length-dependent behavior of mRNA QC, we demonstrated that, it is more challenging for Tpr to retain short aberrant mRNAs compared to longer ones. The reason for this length-dependency is the more time that longer mRNAs spend in the vicinity and inside the nuclear basket to form their optimal configuration for export. As a result, Tpr is able to capture long aberrant mRNAs, while short aberrant mRNAs can quickly escape this step as they do not require a compaction in their size. Interestingly, it has been recently shown that flow-driven translocation of polymers through nanochannels is also length-dependent^48^. Moreover, we demonstrated that the affinity between Tpr and RBPs is optimized to efficiently retain aberrant mRNAs. This interesting finding stresses on the significant role of binding affinities in such a mechanism that is primarily driven by stochastic interactions.

As mentioned before, our model is a minimal system for the QC of mRNAs and we have primarily explored the retention model. There are several other aspects to this process as well. However, we argue that inclusion of other factors will further refine the performance of mRNA QC. In fact, given the imperfect retention of aberrant mRNAs in some cases in our simulations, we expect other factors to be involved to improve the performance of the system. In our model, Tpr has the same affinity to RBPs bound to either normal or aberrant mRNAs, because it is not known whether phosphorylation state of RBPs affect their interaction with the Tpr. However, in the case that Tpr has different affinities to RBPs bound to normal and aberrant mRNAs, it is expected to interact more strongly with RBPs bound to aberrant mRNAs, which will further improve the performance of our model to retain aberrant mRNAs. As another example, whether Tpr interacts with the complex of RBPs and the export receptor, which is required to evaluate the selection model, has yet to be investigated. Mlp1 (yeast homologue of Tpr) is found in complex with various mRNA export machinery proteins including Mex67 (yeast homologue of NXF1)^49^. Moreover, Mex67 has been reported to indirectly interact with Mlp1^50^. However, whether the indirect interaction between the export receptor and Mlp1 is achieved through RBPs is not known. Nevertheless, in the case this interaction (Tpr with the complex of RBP-export receptor) exists, it is expected that the formation of RBP-export receptor complex modulates the interaction between Tpr with RBPs in order not to disrupt the export of normal mRNAs, which, again, improves the retention performance of the system. Therefore, it is conceivable to suggest that the retention and selection models are both in place to maximize the performance of mRNA QC.

Collectively, we demonstrated that regulation of the affinity of RBPs to the export receptor enables Tpr (Mlp1) to recognize and retain aberrant mRNAs. However, this minimal system was not successful to perfectly retain aberrant mRNAs in some situations, justifying the involvement of several other factors and co-factors in mRNA QC to improve its performance in recognizing aberrant mRNAs.

## Materials and Methods

### Agent-based modelling (ABM): predictive modelling of complex biological systems

ABM is a promising approach to efficiently simulate the spatiotemporal interactions between multiple independent entities (agents) with the objective of assessing their individual effect on the overall system and confirming/predicting subsequent emergent phenomena. ABMs consist of a collection of agents with governing rules that dictate local behaviour and interactions with adjacent agents, resulting in a complex emergent behaviour that may not be obvious from the individual rules. Following the pre-defined environmental conditions, the agents move and locally interact with adjacent agents at each time step. Discretizing the space, on-lattice ABMs consists of a grid of “cells” which could be occupied by one or more agents. Each agent is only aware of agents within its neighbouring cells. The movement and interaction events, specifically defined for different types of agents, are based on some probabilities in conjunction with real-world governing rules for that specific agent. To date, only a few studies have taken advantage of on-lattice ABMs to study different biological systems^51^,^52^,^53^. Our ABM is specifically designed for modelling molecular diffusion, binding, and unbinding with consideration for physical factors such as molecular crowding and steric repulsion^34^,^35^,^36^, and is capable of modelling complex three-dimensional biosystems in a computationally efficient and spatiotemporally detailed fashion. It is worth noting that due to some limitations of mean-field approximation methods, it is inapplicable to use these methods to study mRNA export and QC mechanism (please see Supplementary Information – section C).

### Transforming molecular movement and interactions into probabilities

We use the same method that we previously proposed for movement probability selection based on the following molecular diffusion probability, along with algorithms for realistic consideration of crowding and steric repulsion^34^,^35^,^54^:


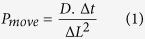


where movement probability of an agent is determined by its diffusion coefficient (D), simulation time step (Δt), and lattice discretization length (ΔL). Reduced probability method is implemented in the model to account for the steric effects of multiple agents occupying individual lattice sites^34^. Detailed derivation of movement probability from diffusion coefficient is explained in the Supplementary Information – section B. Probability selection of binding and unbinding events is based on our more recent work^35^, where we proposed and validated the method to determine probability of binding and unbinding events from kinetic rate constants:


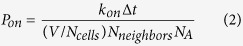



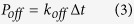


The likelihoods are determined from real-world kinetic rate constants, i.e. k_off_ and k_on_, simulation time step, Δt; system volume, V; number of lattice cells, N_cells_; number of lattice neighbors that each cell has, N_neighbors_; and Avogadro’s number, N_A_. Detailed derivation of probability selection for binding and unbinding events is described in the Supplementary Information – section B.

### ABM system and simulation details

**Figure 2:**
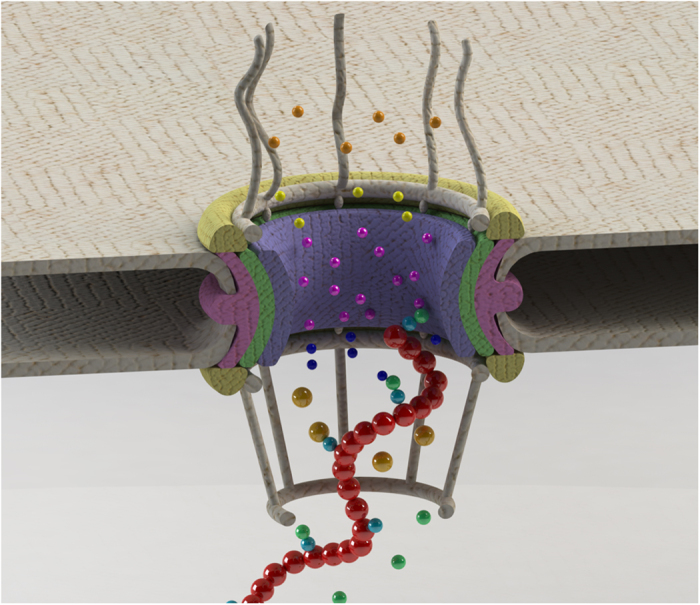
Schematic representation of the overall configuration of the model, including a single nuclear pore complex (NPC) embedded in the nuclear envelope (NE) (a cutaway of the model is presented). NPC scaffold is assumed to be impenetrable to all species, including FG Nups, mRNA (red) and other proteins. FG Nups (yellow, magenta, and blue), Tpr (gold), NXF1/NXT1 (green), RNA-binding proteins (RBPs) (sky blue), and the complex of Dbp5-Gle1-IP6 (orange) are modelled as particle agents in corresponding regions of the environment. The mRNA sequence is modelled as a chain of monomeric agents. For the sake of visual clarity, a small fraction of actual concentrations is shown. Not drawn to scale.

**Figure 3:**
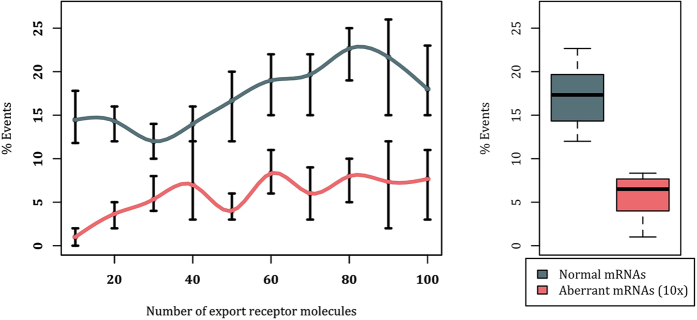
Export rate of an mRNA with 9 RBPs in the absence of the interaction between Tpr and RBPs. Left: Percentage export of mRNA versus export receptor copy number (varied from 10 to 100) for normal affinity between RBPs and NXF1/NXT1 (gray) and 10-fold decreased affinity (pink). For each data point, 100 replicates of the simulation are generated and analysed. The 100-replicate simulations are repeated 3 times. Error bars show the range of values obtained for each data point, while the data point itself is the averaged value among all the 3 repetitions of the 100-replicate simulations. It is clear that, regardless of the export receptor population, aberrant mRNAs cannot reach the normal mRNA export percentage. Right: Boxplot showing average successful export percentage across different NXF1/NXT1 copy numbers. In average, the successful export percentage of the normal mRNA is three times that of the aberrant one.

**Figure 4:**
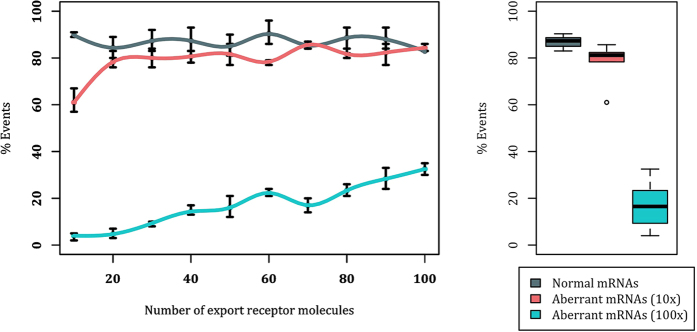
Export percentage of an mRNA with 12 RBPs in the absence of the interaction between Tpr and RBPs . Left: Successful export percentage of mRNA versus export receptor copy number (varied from 10 to 100) for normal affinity between RBPs and NXF1/NXT1 (gray), 10-fold decreased affinity (pink), and 100-fold decreased affinity (cyan). For each data point, 100 replicates of the simulation are generated and analyzed. The 100-replicate simulations are repeated 3 times. Error bars show the range of values obtained for each data point, while the data point itself is the averaged value among all the 3 repetitions of the 100-replicate simulations. Regardless of the copy number of NXF1/NXT1 molecules, except for the first data point, aberrant mRNAs that have 10-fold decreased affinity RBPs are transported as efficient as normal mRNAs. Right: Boxplot showing average successful export percentage across different export receptor copy numbers.

**Figure 5:**
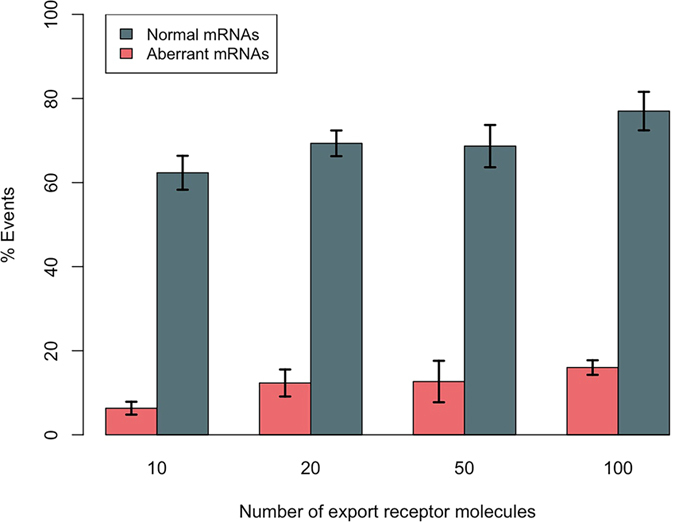
Effect of the interaction between Tpr and RBPs on the export of normal and aberrant mRNAs of length 2.2 kb with 12 RBPs. Four different populations of export receptors are considered inside the nucleus, i.e. 10, 20, 50, and 100 molecules. The interaction between Tpr and RBPs efficiently hinders export of aberrant mRNAs (pink), while allowing proper transport of normal mRNAs (gray).

**Figure 6:**
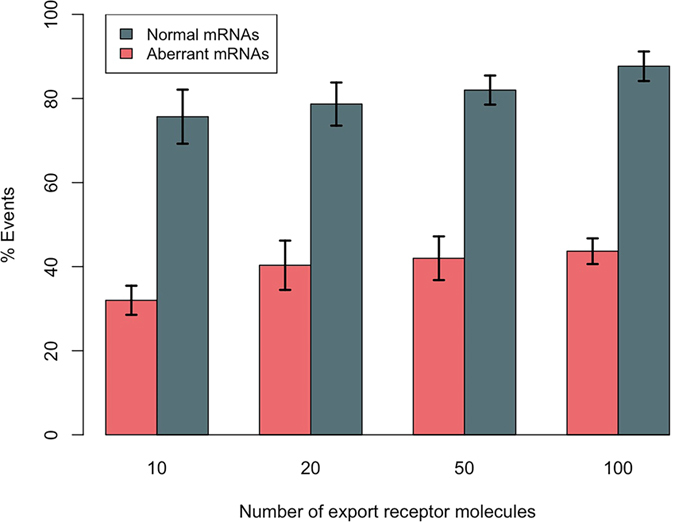
Effect of quality control protein Tpr on the export of normal and aberrant mRNAs of length 500 b with 3RBPs (similar number of export receptor binding sites per unit length as the mRNA simulated in the previous section). Four different populations of export receptors are considered inside the nucleus, i.e. 10, 20, 50, and 100 molecules. Aberrant mRNAs are relatively retained compared to normal mRNAs. However, it is clear that QC mechanism is not as efficient as it is in the case of longer mRNAs (Fig. 5). Therefore, length of mRNA is a determining factor in mRNA QC.

**Figure 7:**
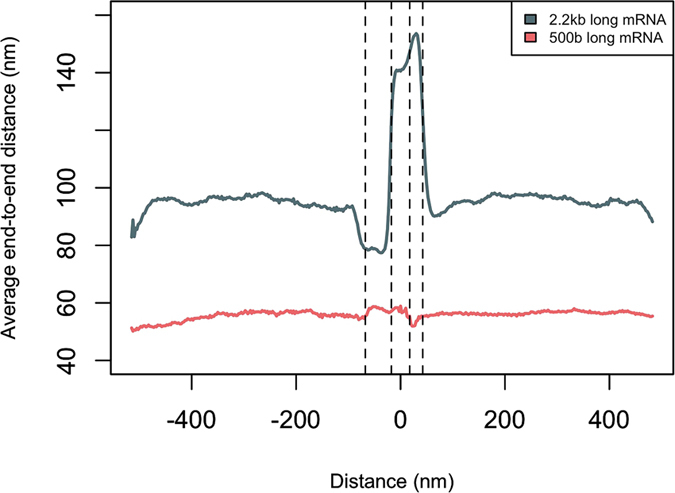
The average end-to-end distance of mRNAs averaged over 100 simulations with respect to their distance from the central channel of the NPC. Two mRNAs are simulated with lengths 500 b and 2.2 kb. The *x*-axis represents the position along the axis perpendicular to the nuclear envelope, with *x* = 0 set at the center of the central channel of the NPC. From left to right, the dashed lines represent the distal edge of the nuclear basket, the nuclear edge of the central channel, the cytoplasmic edge of the central channel, and the distal edge of the cytoplasmic filaments, respectively. The end-to-end distance of the longer mRNA is significantly dropped as it approaches the nuclear basket, to form a more compact conformation to be able to pass through the NPC^36^. The short mRNA, on the other hand, does not show any compaction in size as it reaches the NPC. This difference explains the length-dependent nature of mRNA QC process.

**Figure 8:**
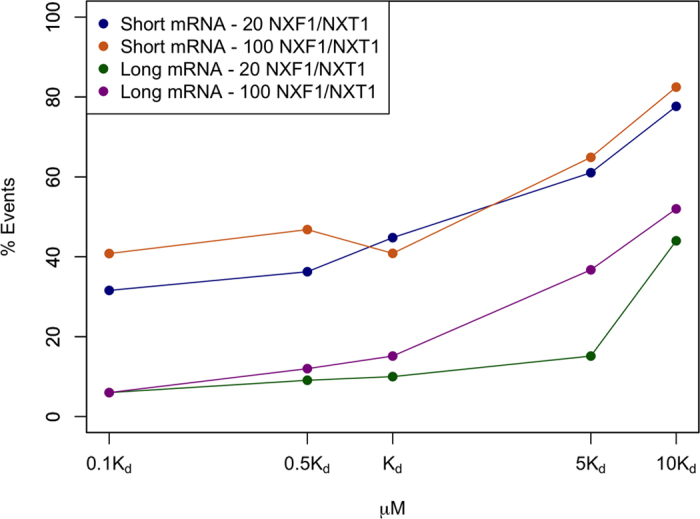
Successful export percentage of aberrant mRNAs with respect to the affinity between Tpr and RBPs. Dissociation constant of Tpr to RBPs is changed from 0.1K_d_ to 10K_d_, where K_d_ is the experimentally measured value of the dissociation constant, to demonstrate how efficient this interaction is in retaining aberrant mRNAs. Two different populations of NXF1/NXT1 dimers (20 and 100) and two different mRNA lengths (500 b and 2.2 kb) are simulated. Dissociation constants smaller than the reported value (~1 μM) almost yield the same export percentage, while larger dissociation constants result in significantly higher export percentage of aberrant mRNAs. Therefore, the ~1 μM dissociation constant is optimized such that, with the weakest interaction possible, it efficiently retains aberrant mRNAs.

Our ABM model is thoroughly verified and validated against other methods as well as *in vitro* and/or *in vivo* results and observations. The validation steps are comprehensively explained in the Supplementary Information – section D. In the model, mRNA is represented as a polymer of bound monomeric agents, which are limited in their movements through the constraint of maintaining connection with their nearest neighbors. These agents are only allowed to move in the diagonal direction into a nearest neighbor’s von Neumann neighborhood to ensure that all movements are of the same length and all movement events of a specific agent type could be accommodated by a single movement probability. The model environment consists of a 42,108-element, three-dimensional lattice composed of cubic elements with dimensions of 5 nm × 5 nm × 5 nm. The lattice size was selected to accommodate the volume associated with the Stokes radius of the largest single-agent species in the system, in this case a collection of nucleotides representing twice the persistence length of the mRNA, or Kuhn length (ranging from ~0.5 to 3 nm^55^,^56^). Additionally, the model allowed for multiple agents of the same or different species type to occupy the same lattice element at any given time, so long as the available volume of a lattice element was not exceeded by agents diffusing into it. Discrete lattice elements belong to one of six region types: cytoplasmic, nuclear membrane, nucleoplasm, cytoplasmic filament periphery, central channel, or nuclear basket. The cytoplasmic region contains a high concentration of Dbp5 in complex with Gle1 and IP6 while the nucleoplasm in each simulation contained a single mRNA, discretized into a number of agents (Fig. 2). The 35 nm-thick nuclear membrane which partitions the two compartments is impermeable to all agent types and contains a single nuclear pore with a diameter of 30 nm at the center and 50 nm at the peripheries. The cytoplasmic filament periphery consists of a 50 nm diameter region that extends 30 nm into the cytoplasm while the nuclear basket is composed of a basket shaped region that extends 55 nm into the nucleoplasm^57^,^58^. Due to the eight-fold symmetry of the NPC^58^,^59^, the cytoplasmic periphery, central channel, and nuclear basket each contain 24, 80, and 32 agents, respectively, representing the distribution of FG Nups^60^. In addition to these FG agents, non-FG agents are added to the channel to represent regions of the Nups that lack affinity for transport receptors but play a role in sterically repelling molecules, with the sum of the volume of these Nups corresponding to experimentally reported volumes^60^ (please refer to^61^,^62^,^63^ for recent comprehensive reviews on NPC and^64^,^65^,^66^,^67^,^68^ for detailed analysis of the behaviour of FG Nups). The collection of agents representing the mRNA is free to diffuse throughout the system while FG agents and non-FG agents are restricted to movement within their respective pore regions in order to maintain the permeability barrier. Figure 2 schematically shows the model used in this study.

The nucleus contains a certain number of NXF1/NXT1 export receptors, which can bind to RBPs bound to the mRNA sequence. RBPs are evenly distributed along the mRNA sequence according to the desired density. The affinity between different RBPs and NXF1/NXT1 was assumed to be the same. This affinity was taken from a recent *in vitro* study, which was reported to be approximately 0.09 μM^42^. Accuracy of the affinity to mimic mRNA export is evaluated (please see the Supplementary Information – section D). Population of NXF1/NXT1 inside the nucleus was varied across simulations. In order to represent aberrantly processed mRNAs, the affinity between RBPs and NXF1/NXT1 was decreased by 10 and/or 100-fold (please see the Results section for the reason for decreased affinities). These values are chosen to be much higher than the variations in the measured affinity for different lengths of mRNAs^42^. For each configuration, 100 replicate simulations were generated and analyzed. Where necessary, the 100-replicate simulations were repeated three times. Each simulation contained a single mRNA with a random initial configuration inside the nucleoplasm. Each simulation was carried out for the duration of 20 seconds using a timestep of 2.5 μs. In our model, we have assumed that Tpr has the same interaction with all RBPs bound to the mRNA sequence. The affinity of the interaction, i.e. ~1 μM, is taken from an *in vitro* study^23^. Moreover, Tpr only interacts with RBPs that are not bound to export receptor, because there is no experimental evidence on the interaction between the complex of RBP-NXF1/NXT1 and Tpr. This is further discussed in the Discussion section. The location of the 5′ and 3′ termini along with the number of mRNA monomers passed the NPC were tracked over the course of the simulation. The trajectories were analyzed to determine the fraction of successful transports per configuration. mRNAs that have completely passed the NPC were considered successful transport events. As we previously showed that a double-tag approach provides more realistic results compared to single-tag labelling^36^, here, a double-tag approach is adopted to track mRNAs.

## Additional Information

**How to cite this article**: Soheilypour, M. and Mofrad, M.R. K. Regulation of RNA-binding proteins affinity to export receptors enables the nuclear basket proteins to distinguish and retain aberrant mRNAs. *Sci. Rep.* **6**, 35380; doi: 10.1038/srep35380 (2016).

**Publisher’s note:** Springer Nature remains neutral with regard to jurisdictional claims in published maps and institutional affiliations.

## Supplementary Material

Supplementary Information

## Figures and Tables

**Figure 1:**
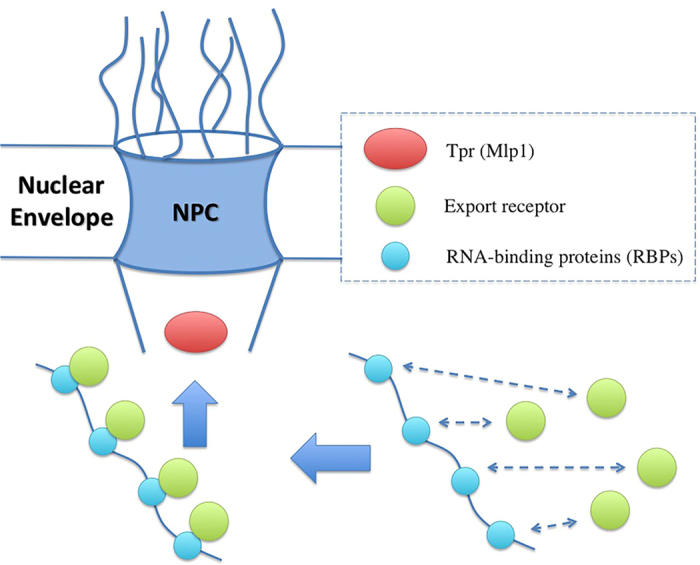
The currently proposed model for mRNA QC at the nuclear basket. RNA-binding proteins (RBPs), including Nab2, Npl3, Hrb1, Gbp2, 9G8, SRp20, and ASF/SF2, are suggested as adapters for recruitment of the export receptor heterodimer (NXF1/NXT1 or Mex67/Mtr2). On the other hand, nuclear basket protein Tpr (Mlp1 in yeast) interacts with RBPs and, as a checkpoint, verifies the maturity of the mRNPs. However, it is still unclear how the emergent behaviour of this system yields an efficient retention of aberrant mRNAs.
