# Supplementary material for: Regulation of RNA-binding proteins affinity to export receptors enables the nuclear basket proteins to distinguish and retain aberrant mRNAs
Source: Sci Rep. 2016 Nov 2;6:35380. doi: 10.1038/srep35380 (PMC5090210; doi:10.1038/srep35380)
Supplement: Supplementary Information [file srep35380-s1.pdf]

Supplementary Information

for

Regulation of RNA-binding proteins affinity to export receptors enables the nuclear basket proteins to distinguish and retain aberrant mRNAs

M. Soheilypour[1], M. Mofrad*[1]

[1] Department of Bioengineering, University of California Berkeley, Berkeley, CA, 94720, USA

* Corresponding author

Tel: +1-510-643-8165

Fax: +1-510-642-5835

Email: mofrad@berkeley.edu

A. Limitations of the model

Quality control (QC) of mRNAs is a sophisticated pathway with a multitude of proteins involved, presumably to optimize the emergent behavior of the system. Considering the complex nature of this process, assumptions were necessary to develop a minimal model best fitted to study this process. In this work, we mainly consider the most influential factors to explore the dynamics of the system and shed light on the mechanism of mRNA export and QC. Some of the factors are not included in the model because they are either suggested to have no direct role in nuclear-associated mRNA QC or are dispensable and act as cofactors (such as Aly/Yra1 [1]). For instance, the evolutionary conserved transcription/export (TREX) complex [2,3], is not included in the model because despite the role of TREX complex in mRNA export, deletion of one of its elements (Mft1) as well as mutation of another element (Yra1) have no effect on the leakage of unspliced transcripts, implying that TREX has no direct role in QC of mRNAs [4].Nuclear basket-associated factors other than Tpr are not included as well, because Tpr (Mlp1/Mlp2 in yeast) is suggested to be the main contributor to QC of mRNAs [4–6], while others have no direct role. In our model, we do not consider the degradation mechanism of aberrant mRNAs, in which aberrant mRNAs are marked by yeast Trf-Air-Mtr4 polyadenylation complex (TRAMP) for degradation [4,7,8], and will be subsequently degraded by nuclear exosome [9]. In addition, NXF1/NXT1 is a shared resource inside the nucleus, recruited by all different types of transcripts and proteins being exported out of the nucleus through all the NPCs embedded in the nuclear envelope (NE). Since our simulations consist of a single NPC, we used "number of molecules per NPC" as the concentration of NXF1/NXT1. Nevertheless, we expect that the trends observed in this study will hold valid under *in vivo* conditions. Moreover, RBPs are uniformly distributed along the mRNA, while it is still unclear how they are distributed *in vivo*. However, our results demonstrate that our proposed model is capable of capturing the dynamics of mRNA export and QC with a reasonable resolution and accuracy and predict the emergent behavior of the system in such a way that is not easily tractable via experimental set ups or other computational techniques.

B. Derivation of agent-based modeling (ABM) probabilities from real-world properties

Relating Diffusion Coefficients to ABM Movement Probabilities[10]

Fick's second law relates the effect of diffusion on the concentration field of particles over time[11]. We can express this relationship in terms of the probability of discretized cells being occupied rather than concentration, Eq. (1), by considering the relationship between concentration and the probability of a cell being occupied by an agent, Eq. (2).

$$\frac{\partial P^{occ}}{\partial t} = D\frac{\partial^2 P^{occ}}{\partial x^2} \tag{1}$$

$$C = \frac{N_{particles}}{N_{cell}V_{element}N_A} = \frac{P^{occ}}{V_{element}N_A} \tag{2}$$

Note that the variables P^{occ}, D, t, and x in Eq. (1) represent probability of finding an occupied cell, diffusion coefficient, time, and position respectively while the

variables C, $N_{particles}$, N_{cell},$V_{element}$, N_A, and P^{occ} in Eq. (2) represent concentration, number of particles, number of cells, volume of each element, Avagadro's number and the probability of finding an occupied cell respectively.

The diffusion term in Eq. (1) is a factor dependent on temperature of the solvent, size and shape of the particle, and viscosity of the solvent that quantifies the ratio of Brownian forces to drag forces. Factors such as force and velocity are not explicitly calculated in a simple ABM and the coarse discretization of space that limits the direction of movement would make such calculations meaningless. Rather, in an ABM, diffusion can be simulated by assigning a probability of movement to each particle agent. The relation between movement probability and a physically meaningful diffusion coefficient is derived below.

We consider a one-dimensional lattice with discretized segments of length ΔL as shown in FigureS1 to derive the relationship between a physical diffusion coefficient and a movement probability to be used in our ABM. We can define the probability of finding a single particle at position X_n at time $t+\Delta t$ as

$$P_{n,t+\Delta t} = P_{n,t} - P_{n,t}T_R - P_{n,t}T_L + P_{n-\Delta L,t}T_R + P_{n-\Delta L,t}T_L \quad (3)$$

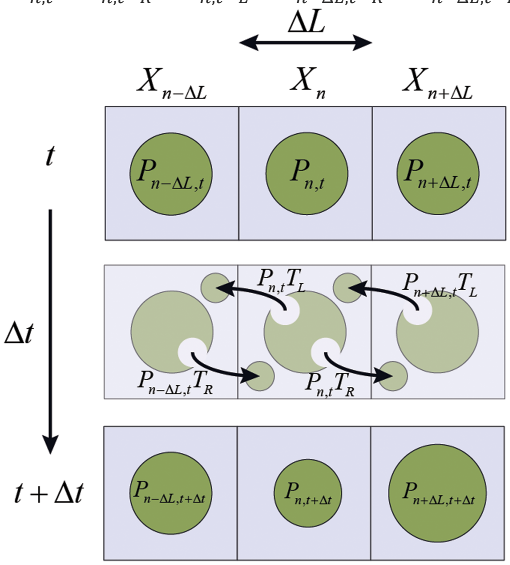

FigureS1: Illustration of movement probabilities in a single dimension. Discretized one-dimensional space with square lattices of length $\Delta \boldsymbol{L}$ depicting how probabilities of particles existing in a cell at time t combined with movement probabilities result in a change in the probability of a particle occupying a cell at time $\boldsymbol{t} + \Delta \boldsymbol{t}$ as outlined by Eq. 3. Note that the circles in each cell do not represent individual particles; rather they qualitatively represent probabilities of a particle residing in that cell.

Where $P_{n,t}$, $P_{n-\Delta L,t}$ and $P_{n+\Delta L,t}$ represent the probability of finding the particle at position X_n, $X_{n-\Delta L}$ and $X_{n+\Delta L}$ respectively at time t; T_R and T_L represent the probability of the particle moving to the right or left respectively. Note that unless otherwise noted, all probability terms represent the probability of the respective cell being occupied. Eq. (3) states that the probability of a particle being found at X_n at time $t+\Delta t$ can be determined based on the probability that the particle was initially in that position and remained there (*first term*) minus the probability that the particle started in that position and moved to either the right or left cells (*second and third term*) plus the probability that the particle

was initially to the left or right of that cell and moved to the right or left respectively (*fourth and fifth term*).

Taylor expansion of the terms in Eq. (3) as $\Delta t, \Delta L \rightarrow 0$ gives the following relationship:

$$\frac{\partial P_{n,t}}{\partial t} = P_{move} \frac{(\Delta L)^2}{\Delta t} \frac{\partial^2 P_{n,t}}{\partial x^2} \tag{4}$$

This assumes that movement probabilities in both directions are equal (T_L=T_R=P_{move}). Eq. (4) relates how the transition probability affects the spatial distribution of particles with time, similar to Eq. (1). Thus we can relate diffusion (*D*) to movement probability (P_{move}), using discretization length (*ΔL*) and time (*Δt*) as shown in Eq. (5) for diffusion of a particle on a discrete cubic lattice.

$$D = P_{move} \frac{(\Delta L)^2}{\Delta t} \qquad \Delta t \rightarrow 0, \Delta L \rightarrow 0 \tag{5}$$

Relating molecular binding and unbinding events to ABM probabilities[12]

The simple case of the molecular unbinding event, which is representative of a first-order unimolecular reaction, can be modeled in an ABM using an unbinding probability, for which derivation of the relationship between kinetic rate constant and probability is trivial. The reversible binding of two molecules *A* and *B* is given in Eq. (6), followed by the rate law for the unbinding event as a function of number of bound molecules within the volume of interest (V):

$$A + B \rightleftharpoons AB \ (k_{on} \ and \ k_{off}) \tag{6}$$

$$\frac{\partial}{\partial t}\left(\frac{N_{AB}}{V.N_{Avogadro}}\right) = -k_{off} \frac{N_{AB}}{V.N_{Avogadro}} \tag{7}$$

Change in the number of bound molecules (∂N_{AB}) is a function of elapsed time (δT), kinetic rate constant (K_{off}) and initial number of bound molecules (N_{AB}) (Eq. (8)). Subsequently, the probability that two bound molecules become unbound is independent of interaction with other molecules; this unbinding probability (P_{off}) is shown in Eq. (10) in the limit of very small Δt.

$$\partial N_{AB} = -\partial t. k_{off} N_{AB} \tag{8}$$

$$\Delta N_{AB} = -P_{off} N_{AB} \tag{9}$$

$$P_{off} = k_{off} \Delta t \tag{10}$$

For molecular binding of two molecules, representative of a second order reaction between adjacent molecules on a lattice - factors such as number of lattice neighbors and lattice size must be considered. This relationship can be derived from the second order rate law as a function of number of unbound molecules within the volume of interest as shown in Eq. (11).

$$\frac{\partial}{\partial t}\left(\frac{N_{AB}}{V.N_{Avogadro}}\right) = k_{on}\frac{N_A}{V.N_{Avogadro}}\frac{N_B}{V.N_{Avogadro}} \tag{11}$$

Similar to the case of unbinding, change in number of bound molecules can be expressed as a function of binding events in terms of number of unbound molecules ($N_A$and N_B) in the volume and their binding kinetic rate constant (k_{on}) (Eq. (12)) as well as a function of binding probability (P_{on}) and the probability of finding neighboring binding molecules within the lattice system ($P_{A-B-neighboring}$), Eq. (13).

$$\partial N_{AB} = \frac{k_{on}N_A N_B \partial t}{V.N_{Avogadro}} \tag{12}$$

$$\Delta N_{AB} = P_{on}P_{A-B-neighboring} \tag{13}$$

The likelihood of finding two unbound agents, *A* and *B*, neighboring each other on the lattice ($P_{A-B-neighboring}$) is a function of the number of unbound *A* molecules (N_A), number of unbound *B* molecules (N_B), number of lattice cells (N_{Cells}), where number of lattice cells in the system is much larger than the number of unbound molecules and the number of lattice neighbors each cell has ($N_{Neighbors}$), Eq. (14).

$$P_{A-B-neighboring} = \frac{N_A N_B}{N_{Cells}}N_{Neighbors} \tag{14}$$

Subsequently, the probability of a binding event between two neighboring molecules (P_{on}) can be derived by solving for the likelihood of neighboring binding molecules in the system ($P_{A-B-neighboring}$) and combining with Eqs. (12) and (13) as shown in Eq. (15).

$$P_{on} = \frac{k_{on}\Delta t}{V/_{N_{Cells}}.N_{neighbors}.N_{Avogadro}} \tag{15}$$

The general form of the probability of binding provided in Eq. (15) is valid for two molecules of different types. For binding events consisting of two molecules of the same type, the probability is reduced by half. Furthermore, Eq. (15) represents the case where the lattice is restricted to containing a single molecule per cell. In the case where multiple smaller molecules can occupy a single cell ($V_A + V_B \leq 1$), a correction factor (α) must be added to the number of neighbors since two unbound agents within the same cell can bind one another. Eq. (16) provides an approximation for the correction factor as a function of the sum of molecular volumes and cell volume.

$$\alpha \approx 1 - \frac{V_A + V_B}{V_{cell}} \tag{16}$$

It should be noted that timestep selection is governed by the smallest of all time scales associated with diffusion or molecular interaction. In other words, the simulation timestep should be selected in a manner so that movement or binding/unbinding event probability does not exceed a value of one.

C. Mean-field methods are not applicable to mRNA export and quality control system

Mean-field approximation methods, such ordinary differential equations (ODEs), are common computational methods used to study biological systems. Stochastic methods and specifically agent-based modeling (ABM) are in general superior to mean-field methods because they account for stochasticity in the system. Moreover, mean-field methods assume a well-mixed system. Besides these general reasons, there are some more specific reasons that mean-field methods are not applicable to mRNA export and quality control system.

1. Mean-field methods assume high number of factors and bulk interactions to model the system. However, in this study, we are dealing with low concentrations and numbers for some of the involved molecules. Moreover, each simulation contains only a single mRNA, which could not be modeled using mean-field methods, as they need high numbers to be able to approximate the effect of individuals by their averaged effect.

2. In mean-field methods, it is not applicable to model polymers such as mRNAs. Since mRNA is a polymeric molecule, entopic effects have a substantial role in the dynamics of mRNA transport through the NPC. The computational studies that are concerned with polymers require modeling them using a method with a finer spatial resolution compared to mean-field methods. As an example, Ledesma-Aguilar et al. [18] used a coarse-grained bead-spring model for polymers, to be able to account for entropic effects of polymer translocation.

There are other minor reasons that make mean-field approximation inapplicable for this study. For instance, export receptors can only bind to specific binding sites on mRNA in a very dynamic manner. We cannot model such a behavior in mean-field methods. As another example, the number of binding sites for export receptors on mRNA and whether they are occupied or not is key to mRNA export. Using mean-field methods, we need to make extra simplifying assumptions (for which no prior knowledge is available) for allowing an mRNA to initiate the export process. Therefore, even overlooking these fundamental reasons and modeling the system using a mean-field would lead to results that are not biologically relevant.

D. Validation of the model

We have validated various aspects of our ABM versus other well-established modeling techniques as well as available experimental results and observations.

<u>Capturing the normal diffusion behavior of molecules</u>[10]

To validate our model for the diffusion of a single particle, we simulated a macromolecule with a Stokes radius of r = 5 nm (diameter = 10 nm) that was free to

diffuse in a solvent in three-dimensional space with a diffusion coefficient of D = 100 mm^2/s. We ran our model for 500,000 time steps with a minimum sampling size of 300 steps, which resulted in a linear relationship between mean square displacement and time (α= 0.9992, R^2= 0.9999), which implies that the model successfully reproduces normal diffusion behavior.

Molecular binding and unbinding[12]

In order to validate our proposed approach to define binding and unbinding probabilities, we modeled a system consisting of an initial concentration of 3mM (2000 molecules in the well-mixed volume) molecules of type A that undergo an irreversible binding event, $A + A \rightarrow B$. We compared our results to the data of the model using a deterministic ordinary differential equation (ODE) for multiple rate constants (FigureS2). The ABM solution reproduces the average behavior of the ODE solution without the unnatural smoothness that is seen in the deterministic model, which confirms its accuracy in modeling binding and unbinding events.

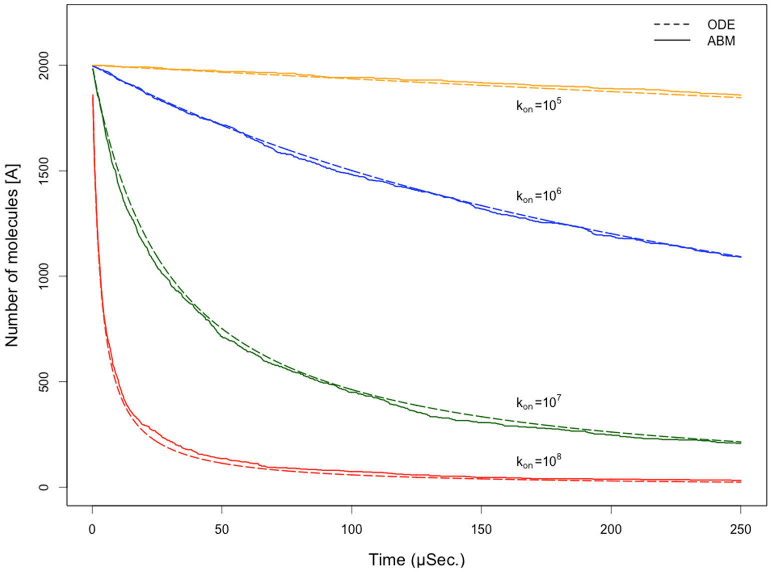

FigureS2: Comparison of ABM and ODE time course data. Comparison of time-course data from an agent-based model of molecular binding to that of the numerical solution of the ordinary differential equation for the same event. Probability selection using the relationship in Eq. 15 produces similar behavior to that of the numerical solution in a well-mixed system at multiple rate constants with the addition of stochasticity that is expected from natural systems.

Nucleocytoplasmic transport of globular cargos through the nuclear pore complex (NPC)[12]

We performed *in silico* experiments to determine the ability of our ABM model to recapitulate experimentally determined, size-dependent permeabilities for globular cargoes including passive cargoes as well as Impβ[13,14]. After the microinjection of non-interacting species agents in the cytoplasm, the NPC was observed to inhibit the influx of larger species while allowing smaller species to diffuse through the pore (FigureS3). Influx rates of non-interacting species with Stokes radii of ~1 nm are on the order of 0.1 s^{-1}, whereas larger species with Stokes radii of >2.5 nm have influx rates of <0.001 s^{-1}. As expected, a reduced influx rate was not observed for larger species that had affinity

for the FG Nups. To test this behavior, we repeated experiments similar to those performed for non-interacting species, replacing the non-interacting species with 2.5 μM labeled Impβ in addition to the steady-state concentration of unlabeled Impβ. The influx rate of Impβ into the nucleus was observed to be 0.367 s^{-1}. This value is comparable with an experimentally measured influx rate of 0.4 s^{-1} for Impβ[13].

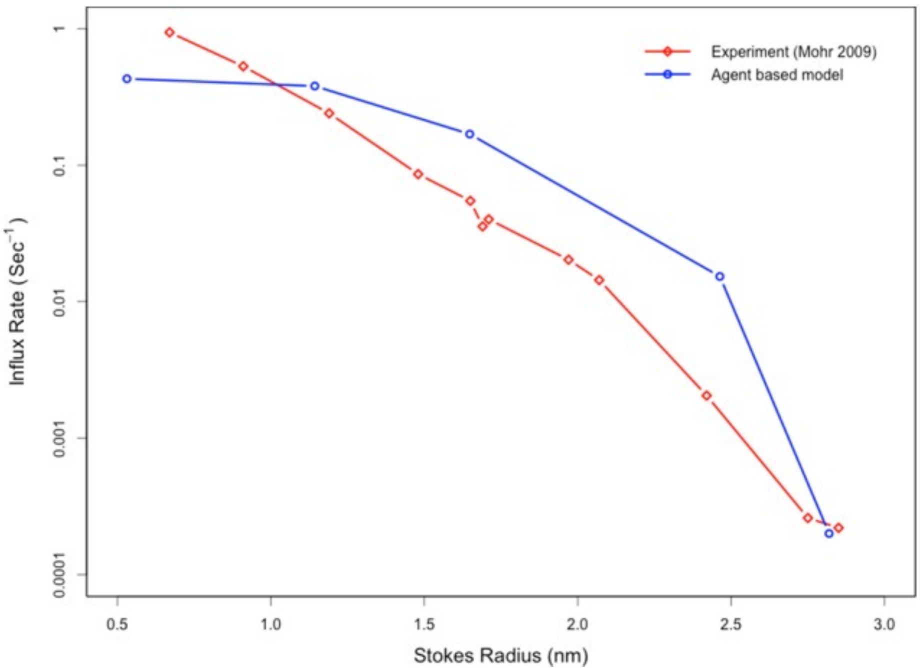

FigureS3: The agent based model recapitulates the experimentally observed size-dependent permeabilities of passive cargos through the nuclear pore. Following a simulated microinjection of non-interacting species in the cytoplasm, the *in silico* pore is observed to inhibit the influx of larger species while allowing smaller species to diffuse through the pore. This is in agreement with previous experimental observations

Movement probability of mRNA

The movement probability of mRNA agents is determined through successive simulations of a 2.2 kb polymer composed of 130 agents over a 12.5 s long simulation. Calculating the mean square displacement of the polymer in the simulations, the results were compared with *in vivo* measurements of mRNA diffusion[15] to determine the corresponding effective diffusion coefficient of ~0.01 $\mu m^2/s$[16].

Validation of the affinity of RNA-binding proteins (RBPs) to the export receptor

In order to verify that the affinity between RBPs and the export receptor is accurate enough, we simulated a 2.2 kb mRNA with the average number (9) of RBPs as binding sites for the export receptor. Our simulations demonstrated that the utilized affinity between RBPs and the export receptor yields the same percentage of successful export events as previously reported from *in vivo* experiments, i.e. ~%20 for an mRNA of the same length[17] (Figure 1). Therefore, the affinity is accurate enough to simulate the indirect interaction between mRNA and the export receptor in our model.

References:

1. Tutucci, E. & Stutz, F. Keeping mRNPs in check during assembly and nuclear export. *Nat. Rev. Mol. Cell Biol.* **12,** 377–84 (2011).
2. Reed, R. & Cheng, H. TREX, SR proteins and export of mRNA. *Curr. Opin. Cell Biol.* **17,** 269–73 (2005).
3. Katahira, J. mRNA export and the TREX complex. *Biochim. Biophys. Acta* **1819,** 507–13 (2012).
4. Hackmann, A. *et al.* Quality control of spliced mRNAs requires the shuttling SR proteins Gbp2 and Hrb1. *Nat. Commun.* **5,** 3123 (2014).
5. Green, D. M., Johnson, C. P., Hagan, H. & Corbett, A. H. The C-terminal domain of myosin-like protein 1 (Mlp1p) is a docking site for heterogeneous nuclear ribonucleoproteins that are required for mRNA export. *Proc. Natl. Acad. Sci. U. S. A.* **100,** 1010–5 (2003).
6. Fasken, M. B., Stewart, M. & Corbett, A. H. Functional significance of the interaction between the mRNA-binding protein, Nab2, and the nuclear pore-associated protein, Mlp1, in mRNA export. *J. Biol. Chem.* **283,** 27130–43 (2008).
7. Sakharkar, M. K., Chow, V. T. K. & Kangueane, P. Distributions of exons and introns in the human genome. *In Silico Biol.* **4,** 387–93 (2004).
8. Porrua, O. & Libri, D. RNA quality control in the nucleus: The Angels' share of RNA. *Biochim. Biophys. Acta - Gene Regul. Mech.* **1829,** 604–611 (2013).
9. Chlebowski, A., Lubas, M., Jensen, T. H. & Dziembowski, A. RNA decay machines: the exosome. *Biochim. Biophys. Acta* **1829,** 552–60 (2013).
10. Azimi, M., Jamali, Y. & Mofrad, M. R. K. Accounting for diffusion in agent based models of reaction-diffusion systems with application to cytoskeletal diffusion. *PLoS One* **6,** e25306 (2011).
11. Fick, A. On liquid diffusion. *J. Memb. Sci.* **100,** 33–38 (1995).
12. Azimi, M. & Mofrad, M. R. K. Higher nucleoporin-Importinβ affinity at the nuclear basket increases nucleocytoplasmic import. *PLoS One* **8,** e81741 (2013).
13. Riddick, G. & Macara, I. G. A systems analysis of importin-{alpha}-{beta} mediated nuclear protein import. *J. Cell Biol.* **168,** 1027–1038 (2005).
14. Mohr, D., Frey, S., Fischer, T., Güttler, T. & Görlich, D. Characterisation of the passive permeability barrier of nuclear pore complexes. *EMBO J.* **28,** 2541–53 (2009).
15. Mor, A. *et al.* Dynamics of single mRNP nucleocytoplasmic transport and export through the nuclear pore in living cells. *Nat. Cell Biol.* **12,** 543–52 (2010).
16. Azimi, M., Bulat, E., Weis, K. & Mofrad, M. R. K. An agent-based model for mRNA export through the nuclear pore complex. *Mol. Biol. Cell* **25,** 3643–53 (2014).
17. Siebrasse, J. P., Kaminski, T. & Kubitscheck, U. Nuclear export of single native mRNA molecules observed by light sheet fluorescence microscopy. *Proc. Natl. Acad. Sci. U. S. A.* **109,** 9426–31 (2012).
18. Ledesma-Aguilar, R., Sakaue, T. & Yeomans, J. M. Length-dependent translocation of polymers through nanochannels. *Soft Matter* **8,** 1884–1892 (2012).
